# Klotho’s Impact on Cardiovascular Disease, Fractures, and Mortality in Hemodialysis

**DOI:** 10.1016/j.ekir.2025.07.032

**Published:** 2025-07-30

**Authors:** Akio Nakashima, Kazuhiko Kato, Arisa Kobayashi, Rena Kawai, Yuriko Shibata, Saya Tanimoto, Chiharu Aizawa, Ichiro Ohkido, Takashi Yokoo

**Affiliations:** 1 Division of Nephrology and Hypertension, Department of Internal Medicine, The Jikei University School of Medicine, Tokyo, Japan

**Keywords:** cardiovascular disease, FGF23, fracture, hemodialysis, Klotho

## Abstract

**Introduction:**

Klotho, an aging-suppressor protein, has been shown to promote cardiovascular and bone health in animal models of chronic kidney disease (CKD). However, limited data exist on its role in clinical outcomes among patients undergoing hemodialysis. This study aimed to investigate the association between soluble Klotho (sKlotho) levels and cardiovascular disease (CVD) events, fractures, and all-cause mortality in this population.

**Methods:**

We enrolled 1241 patients on hemodialysis from multiple medical institutions, with a median follow-up of 39 months. The primary outcome was a composite of CVD events, fractures, and all-cause mortality.

**Results:**

The median sKlotho concentration was 325.6 pg/ml (interquartile range [IQR]: 248.9–434.4 pg/ml). During the follow-up, 436 CVD and 100 fracture events were recorded, along with 228 deaths. Patients in the lowest quartile of sKlotho had significantly higher risks of CVD events (hazard ratio [HR] = 1.76; 95% confidence interval [CI]: 1.20–2.60), fractures (HR = 1.99; 95% CI: 1.01–3.91) and mortality (HR = 1.74; 95% CI: 1.00–3.03) than those in the highest quartile.

**Conclusion:**

These findings suggest that low serum sKlotho levels are strongly associated with poor cardiovascular and skeletal outcomes and increased mortality in patients on hemodialysis. This study highlights the potential utility of sKlotho as a biomarker for risk stratification in this high-risk population.

## Graphical abstract

**Figure:**
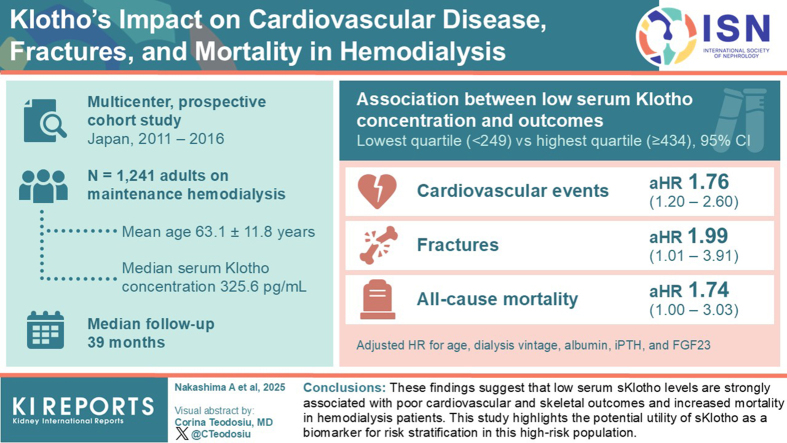

Patients with CKD face a significantly elevated risk of mortality, with CVD being the leading cause of death in this population.^1^ Key drivers of CVD in CKD include hyperphosphatemia and elevated levels of fibroblast growth factor-23 (FGF23), and recent studies have highlighted the role of the Klotho protein in mitigating these risks.^2^^,^^3^ Klotho, encoded by the *Klotho* antiaging gene, serves as a cofactor for FGF23 and regulates phosphorus and vitamin D metabolism.^4^, ^5^, ^6^ This protein exists in 2 forms, namely membrane-bound and sKlotho, with the latter exerting antiaging effects through various pathways, including modulation of insulin signaling, regulation of the renin-angiotensin system, and protection against oxidative stress.^7^

Experimental models have demonstrated that administering sKlotho to Klotho-deficient mice prevents cardiac hypertrophy and increases survival.^8^^,^^9^ Clinically, sKlotho levels have been inversely correlated with left ventricular mass,^10^ and reduced Klotho expression is associated with endothelial dysfunction, oxidative stress, and vascular calcification.^11^ Because sKlotho is predominantly secreted by the kidneys, patients on dialysis often exhibit reduced serum sKlotho levels.^12^ Despite this, limited studies have examined the relationship between sKlotho and CVD events in the dialysis population.

In addition, an association between Klotho and bone homeostasis has been reported. In Klotho-deficient mice, abnormal distribution of bone matrix proteins and impaired bone mineralization have been observed.^13^ Mechanistic studies suggest that sKlotho influences bone metabolism via the Wnt/β-catenin pathway,^14^ and clinical investigations have shown that decreased sKlotho levels are associated with reduced bone mass.^15^^,^^16^ However, there remains a paucity of studies examining the direct relationship between sKlotho levels and clinical fracture events in patients on dialysis.

Previous studies exploring sKlotho levels and clinical outcomes in patients on dialysis were limited by small sample sizes and often did not account for FGF23 levels, which may influence these outcomes. Furthermore, few studies have investigated the association between sKlotho and clinical events such as fractures, CVD, and mortality in a comprehensive manner. Therefore, this study aimed to investigate the relationship between serum sKlotho levels, CVD events, clinical fracture events, and all-cause mortality in a large cohort of patients undergoing hemodialysis.

## Methods

### Study Design

This multicenter prospective cohort study recruited stable patients on hemodialysis from 15 medical institutions in Tokyo, Kanagawa, Saitama, and Chiba, Japan.^17^, ^18^, ^19^ Baseline data were collected from May 1, 2011 to March 31, 2012, with patients followed-up with until June 1, 2016. The inclusion criteria were age > 20 years, a dialysis vintage ≥ 3 months, and regular hemodialysis sessions 3 times a week (3–5 hours/session). The exclusion criteria included patients with acute gastrointestinal bleeding, acute coronary syndrome (defined as acute heart failure, myocardial infarction, or unstable angina), or liver dysfunction at baseline. Patients with recent infections or antibiotic prescriptions were also excluded. The study was approved by the ethics committee at Jikei University School of Medicine and the institutional review boards of all participating hospitals (31-194 9693). All the study procedures adhered to the Declaration of Helsinki and written informed consent was obtained from all participants.

### Data Collection

Patient demographics (age, sex, dialysis vintage, and primary kidney disease) and medical history were obtained from medical records. Medication data, including the use of antiplatelet agents, vitamin K antagonists, phosphate binders, vitamin D receptor agonists, cinacalcet, sevelamers, lanthanum, bixisalomers, calcium-containing phosphate binders, antihypertensive medications, beta-blockers, or statins were collected from prescription data. Comorbidities and medications were confirmed through chart review and standardized interviews at baseline.

Blood samples were collected at study entry, before the hemodialysis session, following the longest interdialytic interval. Routine biochemical measurements included serum sodium, potassium, phosphorus, calcium, magnesium, albumin, blood urea nitrogen, alkaline phosphatase, creatinine, hematocrit, intact parathyroid hormone, and C-reactive protein. The delivered dialysis dose was measured using single-pool Kt/V.

Serum for measuring sKlotho and FGF23 was stored at −80 °C immediately after collection. SKlotho was measured within 1 month of collection using 1 aliquot of serum stored at −80 °C. The serum measured this time was the first one measured after freezing, and no serum that had been repeatedly frozen and thawed was used. sKlotho levels were measured using a sandwich enzyme-linked immunosorbent assay (IBL International, Tokyo, Japan). The measurements were conducted in duplicate. This enzyme-linked immunosorbent assay had a sensitivity of 6.15 pg/ml and an intra-assay coefficient of 18%. Serum intact FGF23 levels were measured using an enzyme-linked immunosorbent assay (KAINOS, Tokyo, Japan). In this study, we measured intact FGF23 rather than c-terminal FGF23. FGF23 was measured at the same time as sKlotho using the same serum.

### Outcomes

Clinical outcomes were prospectively recorded by investigators blinded to patient data. The primary outcomes were major CVD events, including cardiovascular death, sudden death, ischemic heart disease requiring revascularization, heart failure requiring hospitalization, and cerebrovascular events (e.g., cerebral hemorrhage, infarction, or peripheral artery disease requiring bypass operation or stenting). Cerebral infarction was defined as a sudden onset of nonocclusive and focal neurologic deficits persisting for > 24 hours. Peripheral artery disease was defined as tissue necrosis, lower limb amputation, or revascularization procedure for a peripheral artery. The secondary outcomes included clinical fracture events and all-cause mortality. In this study, the incidence of clinical fractures was collected from the medical records. We did not include patients with asymptomatic fractures detected by imaging or annual height loss. In all analyses, we censored follow-up at loss to follow-up, renal transplantation, or at the end of the study.

### Statistical Analysis

Non-ormally distributed data are presented as medians with IQRs, whereas normally distributed data are reported as mean ± SD. Binary data are summarized as numbers and percentages. sKlotho concentrations were divided into quartiles and differences between groups analyzed using analysis of variance or the Kruskal–Wallis test as appropriate. Nominal variables were analyzed using the chi-square (χ^2^) test. To investigate the association between sKlotho levels and CVD events, univariate and multivariate Cox regression analyses were conducted, with results expressed as HRs and 95% CIs. Model 1 included adjustments for age, sex, dialysis vintage, body mass index, systolic blood pressure, diabetes mellitus, hemoglobin, albumin, creatinine, potassium, C-reactive protein, β2 microglobulin, angiotensin-converting enzyme inhibitor or angiotensin II receptor blocker (ARB) use, history of CVD, atrial fibrillation, and Kt/V. Model 2 added adjustments for calcium, phosphate, magnesium, intact (i) parathyroid hormone (PTH) (iPTH), FGF23, and history of percutaneous ethanol injection therapy or parathyroidectomy. Model 3 further adjusted for active vitamin D analogs, lanthanum, sevelamer, bixisalomer, and cinacalcet use. We used competing risk models for the analysis and set death from CVD as a competing risk event. The association between sKlotho levels and all-cause mortality was analyzed using the same method and analysis models used for CVD events. For the analysis of fracture events, we used Cox hazard regression analysis and set model 1, adjusted for age, dialysis vintage, history of fracture, and history of percutaneous ethanol injection therapy or parathyroidectomy. Model 2 was defined as model I plus albumin, beta 2 macroglobulin, magnesium, and FGF23; and model 3 as model 2 plus iPTH and cinacalcet. Competing risk models were used for fracture analysis. Following previous studies, explanatory factors for multivariate analysis were selected to include patient background, nutritional status, medical history, and CKD-mineral and bone disorder factors including calcium, phosphorus, and PTH.^20^, ^21^, ^22^ Kaplan–Meier curves were created for CVD, fracture, and mortality, and the log-rank test was performed. An adjusted restricted cubic spline curve with 3 knots was generated to show the nonlinear association between CVD events, fracture events, and all-cause mortality. sKlotho as a continuous variable was examined using a fully adjusted model. At the outset of the study, group stratification for the subgroup analysis of CVD events was planned using age (65 years), history of CVD, diabetes mellitus, serum phosphorus concentration (6.0 mg/dl), serum iPTH concentration (180 pg/ml), FGF23 (5022 pg/ml), cinacalcet use, and vitamin D analog use by the study population. Statistical significance was set at *P* < 0.05. All statistical analyses were performed using STATA software version 16.0; (STATA Corp., College Station, TX).

## Results

### Patient Characteristics and sKlotho Concentrations

After excluding patients with insufficient blood samples, clinical data, or missing outcomes, a total of 1241 out of 1350 were included in the final analysis. The median sKlotho concentration was 325.6 pg/ml (IQR: 248.9–434.4 pg/ml). sKlotho levels were divided into quartiles: quartile 1 (< 249 pg/ml), quartile 2 (249–326 pg/ml), quartile 3 (326–434 pg/ml), and quartile 4 (≥ 434 pg/ml). Patient characteristics by sKlotho quartile are summarized in Table 1. The mean age was 63.1 ± 11.8 years, and median dialysis vintage was 84 months (IQR: 39–154 months). The mean serum calcium and phosphorus concentrations were 8.9 ± 0.7 mg/dl and 5.5 ± 1.4 mg/dl, respectively; and median iPTH level was 145 pg/ml (IQR, 83–243 pg/ml). Cinacalcet was used by 391 patients (31.5%), whereas 815 patients (65.6%) received active vitamin D analogs. The median FGF23 level was 5022 pg/ml (IQR: 1535–12,650 pg/ml). There were no significant differences in diabetes mellitus, body mass index, serum albumin, or C-reactive protein levels across the sKlotho quartiles. Similarly, no significant differences in calcium, phosphorus, magnesium, or iPTH levels were observed between the quartiles. However, patients in higher sKlotho quartiles tended to have lower serum FGF23 levels than those in lower quartiles.

**Table 1:** Patient characteristics categorized by serum Klotho concentration

Variable	Quartile of serum Klotho level (pg/ml)								*P*-value
	Quartile 1 (< 249)		Quartile 2 (249–326)		Quartile 3 (326–434)		Quartile 4 (≥ 434)		
Number	307		310		309		315		
Variable									
Age (yrs)	64.3	± 11.6	63.6	± 12.0	62.1	± 12.2	62.5	± 11.3	0.093
Male (%)	220	(71.7)	228	(73.6)	212	(68.6)	210	(66.7)	0.239
Medical history									
CVD (%)	58	(18.9)	68	(21.9)	47	(15.2)	47	(14.9)	0.069
PEIT or PTx (%)	14	(4.6)	29	(9.5)	40	(13.4)	33	(10.8)	0.003
Diabetes mellitus (%)	124	(40.4)	116	(37.4)	107	(34.6)	115	(36.5)	0.516
Dialysis vintage (mo)	75	(34–128)	83	(38–157)	98	(44–176)	90	(37–158)	0.02
Body mass index (kg/m^2^)	21.5	± 4.6	21.6	± 5.2	21.8	± 5.5	22.1	± 5.0	0.509
sBP (mm Hg)	152.2	± 21.2	152.6	± 22.6	151.5	± 22.3	151.3	± 21.5	0.877
dBP (mm Hg)	78.4	± 13.9	79	± 13.8	79.2	± 15.1	80.8	± 13.7	0.168
Hemoglobin (g/dl)	10.5	± 1.0	10.5	± 1.0	10.4	± 1.1	10.5	± 1.1	0.838
Albumin (g/dl)	3.7	± 0.3	3.8	± 0.4	3.8	± 0.4	3.7	± 0.3	0.087
BUN (mg/dl)	63.0	± 14.3	65.9	± 14.2	65.3	± 14.4	66.2	± 13.6	0.022
Creatinine (mg/dl)	11.3	± 3.1	11.8	± 2.9	11.8	± 3.3	11.5	± 3.1	0.124
Sodium (mEq/l)	139	± 2.9	139	± 3.1	139	± 3.0	139.3	± 2.7	0.521
Potassium (mEq/l)	5.0	± 0.7	5.0	± 0.8	5.0	± 0.7	5.0	± 0.7	0.191
CRP (mg/dl)	0.13	(0.05–0.4)	0.13	(0.05–0.35)	0.12	(0.05–0.36)	0.12	(0.05–0.32)	0.953
ACE-I or ARB (%)	179	(58.3)	164	(52.9)	149	(48.2)	142	(45.1)	0.001
CCB (%)	118	(38.4)	140	(45.2)	133	(43.0)	118	(37.5)	0.157
ESA (%)	238	(77.5)	248	(80.0)	232	(75.1)	238	(75.6)	0.451
β2mg (μg/dl)	27.1	(23.7–30.2)	27.1	(24.2–30.5)	27.5	(24.4–31.3)	26.9	(23.6–31.2)	0.285
Kt/V	1.36	(1.2–1.52)	1.35	(1.23–1.52)	1.39	(1.23v1.57)	1.4	(1.23–1.60)	0.119
Calcium (mg/dl)	8.9	± 0.7	9	± 0.6	8.9	± 0.7	8.9	± 0.6	0.257
Phosphate (mg/dl)	5.4	± 1.3	5.6	± 1.4	5.5	± 1.4	5.5	± 1.4	0.247
ALP (IU/ml)	205	(161–262)	211	(167–269)	223	(176–299)	231	(184–309)	< 0.001
Magnesium (mg/dl)	2.6	± 0.5	2.6	± 0.4	2.6	± 0.5	2.6	± 0.4	0.619
iPTH (pg/dl)	140	(77–223)	139	(85–210)	162	(90–254)	150	(90–228)	0.053
25OHD (ng/ml)	14.9	(9.8–19.8)	16.4	(12.1–22.1))	16.7	(11.6–22.7)	15.4	(10.4–20.8)	0.098
FGF23 (pg/ml)	4838	(1518–13,098)	6321	(2436–13,336)	4269	(1234–11,429)	4039	(1510–9748)	0.015
Active vitamin D analogue									0.048
oral (%)	94	(30.6)	97	(31.3)	76	(24.6)	111	(35.2)	0.036
i.v. (%)	106	(34.5)	121	(39.0)	107	(34.6)	103	(32.7)	0.397
CaCO_3_ (%)	229	(74.6)	230	(74.2)	230	(74.4)	226	(71.8)	0.832
Sevelamer (%)	41	(13.4)	68	(21.9)	60	(19.4)	65	(20.6)	0.033
Lanthanum (%)	113	(36.8)	119	(38.5)	125	(40.5)	130	(41.5)	0.636
Bixsalomer (%)	10	(3.3)	7	(2.3)	4	(1.3)	10	(3.2)	0.358
Cinacalcet (%)	92	(30.0)	111	(35.8)	96	(31.1)	92	(29.2)	0.285

25OHD, 25-hydroxyvitaminD; ACE-I, angiotensin-converting enzyme-inhibitors; ALP, alkaline phosphatase; ARB, angiotensin II receptor blocker; BUN, blood urea nitrogen; CCB, calcium channel blocker; CRP, C-reactive protein; CVD, cardiovascular disease; dBP, diastolic blood pressure; ESA, erythropoietin stimulating agent; FGF23, fibroblast growth factor 23; iPTH, intact parathyroid hormone; PEIT, percutaneous ethanol injection therapy; PTx, parathyroidectomy; sBP, systolic blood pressure; β2mg, beta 2 microglobulin.

Data are presented as mean (SD), number (%), or median (interquartile range), as appropriate.

### Multivariate Analysis for sKlotho

Multivariate regression analysis identified several factors associated with sKlotho concentrations (Supplementary Table S1). Significant associations were found between sKlotho levels and age, FGF23, and a history of percutaneous ethanol injection therapy or parathyroidectomy. In addition, the use of cinacalcet and active vitamin D analogs was significantly associated with sKlotho levels. However, no significant differences were observed between sKlotho levels and diabetes mellitus, serum albumin, body mass index, calcium, phosphate, magnesium, or PTH levels.

### sKlotho Concentrations and CVD Events

**Figure 1:**
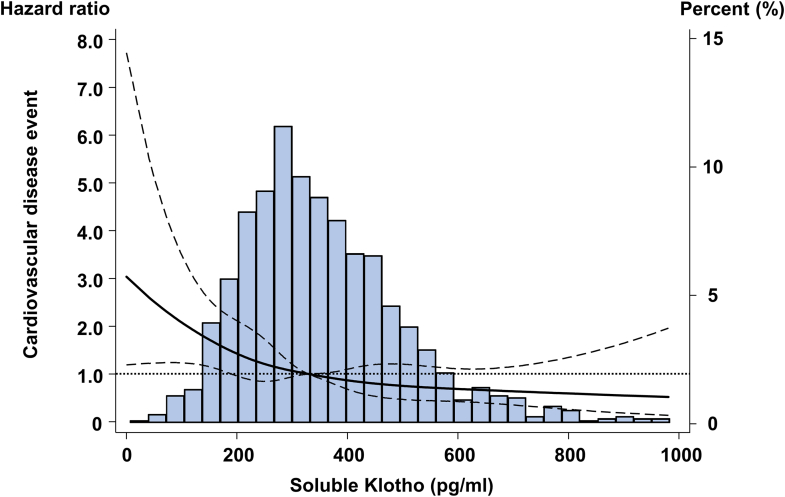
Restricted cubic spline curve of the soluble Klotho levels and cardiovascular disease events.

During the follow-up period, 436 CVD events were recorded, including cardiovascular or sudden death (*n* = 110), ischemic heart disease (*n* = 147), heart failure (*n* = 38), cerebrovascular events (*n* = 81), and peripheral artery disease (*n* = 60). The Kaplan–Meier curves and the restricted cubic spline curve demonstrated that lower sKlotho levels were associated with an increased risk of CVD events (Figure 1, Supplementary Figure S1). Cox proportional hazards regression analysis was performed across unadjusted and adjusted models (Models 1–3) (Table 2, Supplementary Table S2). This association between sKlotho levels and CVD events persisted even after adjusting for additional covariates, including markers of mineral bone disorder in model 2 (HR = 1.79, 95% CI: 1.22–2.63), and the use of phosphate binders, vitamin D analogs, and calcimimetics in model 3 (HR = 1.76; 95% CI: 1.20–2.60). Other significant factors for CVD events included age, history of diabetes, hemoglobin, history of ischemic heart disease, and dialysis vintage.

**Table 2:** Cox hazard regression analysis for cardiovascular disease events, fracture events and all-cause death by serum soluble Klotho concentration

	Quartiles of serum Klotho concentration (pg/ml)							*P*-value
	Quartile 1		Quartile 2		Quartile 3		Quartile 4	
	(< 249)		(249–326)		(326–434)		(≥ 434)	
Participants, *n*	307		310		309		315	
CVD events, *n*	136		116		97		87	< 0.001
Unadjusted HR (95% CI)	1.84	(1.37–2.48)	1.43	(1.06–1.95)	1.15	(0.84–1.59)	Ref	< 0.001
Fully adjusted HR (95% CI)^a^	1.76	(1.20–2.60)	1.14	(0.95–2.10)	0.96	(0.64–1.45)	Ref	< 0.001
Fracture events, *n*	30		27		25		18	< 0.001
Unadjusted HR (95% CI)	1.83	(1.01–3.31)	1.65	(0.91–3.01)	1.49	(0.81–2.72)	Ref	< 0.001
Fully adjusted HR (95% CI)^b^	1.99	(1.01–3.91)	2.04	(1.02–4.07)	1.8	(0.91–3.56)	Ref	< 0.001
All-cause death, *n*	61		70		50		47	< 0.001
Unadjusted HR (95% CI)	1.40	(0.96–2.04)	1.57	(1.08–2.27)	1.09	(0.73–1.62)	Ref	< 0.001
Fully adjusted HR (95% CI)	1.74	(1.00–3.03)	1.92	(1.11–3.36)	1.13	(0.63–2.03)	Ref	< 0.001

CaCO_3_, calcium carbonate; CI, confidence interval; CVD, cardiovascular disease; HR, hazard ratio.

a Cardiovascular events and all-cause death are adjusted for age, sex, dialysis vintage, body mass index, systolic blood pressure, diabetes mellitus, hemoglobin, albumin, creatinine, potassium, C-reactive protein, beta 2 microglobulin, use of angiotensin-converting enzyme-inhibitors or angiotensin II receptor blockers, history of cardiovascular disease, history of atrial fibrillation, Kt/V, calcium, phosphate, magnesium, intact parathyroid hormone, fibroblast growth factor 23, 25-hydroxyvaitamin D, history of percutaneous ethanol injection therapy or parathyroidectomy, active vitamin D analog, lanthanum, sevelamer, bixasalomer, CaCO_3_, and cinacalcet.

b Fracture events are adjusted for age, dialysis vintage, history of fracture, history of percutaneous ethanol injection therapy or parathyroidectomy, albumin, beta 2 microglobulin magnesium, fibroblast growth factor 23, intact parathyroid hormone and cinacalcet.

### sKlotho Concentrations and Clinical Fracture Events

**Figure 2:**
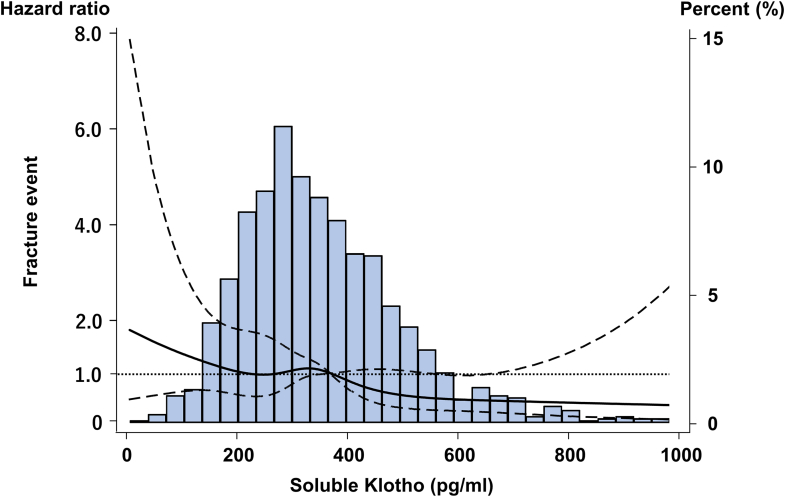
Restricted cubic spline curve of the soluble Klotho levels and fracture events.

A total of 100 clinical fractures occurred during the study, including vertebral fractures (*n* = 27), femoral fractures (*n* = 31), and other fractures (*n* = 42). The Kaplan–Meier curves and restricted cubic spline curve (Figure 2, Supplementary Figure S2) indicated that the risk of clinical fractures was decreased with higher sKlotho levels. In the fully adjusted model (model 3), the lowest sKlotho group (quartile 1) and next lowest group (quartile 2) were significantly associated with an increased risk of fracture events compared with the highest sKlotho group (quartile 1: HR = 1.99, 95% CI: 1.01–3.91; quartile 2: HR = 2.04, 95% CI: 1.02–4.07) (Table 2, Supplementary Table S3). Other significant factors for fracture included age, use of active vitamin D analogues, use of cinacalcet, albumin, and past history of fractures.

### sKlotho Concentrations and All-Cause Mortality

**Figure 3:**
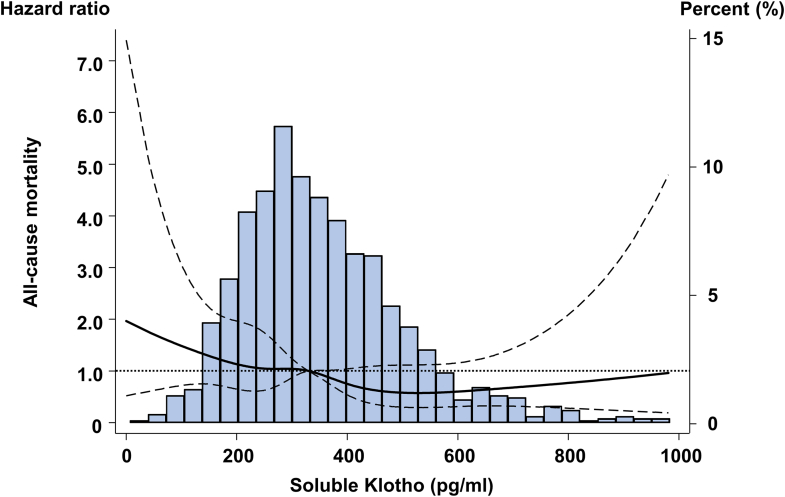
Restricted cubic spline curve of the soluble Klotho levels and all-cause mortality.

During the follow-up period, 228 deaths were recorded, with the following causes: CVD (*n* = 37), sudden death (*n* = 53), cerebrovascular disease (*n* = 20), infectious disease (*n* = 62), malignant disease (*n* = 34), and other or unknown causes (*n* = 22). The Kaplan–Meier curves and restricted cubic spline curve also showed that low sKlotho concentrations increased the risk of all-cause mortality (Figure 3, Supplementary Figure S3). In the fully adjusted model (model 3), both the lowest sKlotho group (quartile 1) and next lowest group (quartile 2) were significantly associated with increased all-cause mortality compared with the highest sKlotho group (quartile 1: HR = 1.74, 95% CI: 1.00–3.03; quartile 2: HR = 1.92, 95% CI: 1.11–3.36) (Table 2, Supplementary Table S4). Other significant factors included age, sex, albumin, use of angiotensin-converting enzyme inhibitor or ARB, beta 2 mg, dialysis vintage, and use of active vitamin D analogues.

### Subgroup Analysis of sKlotho and CVD

**Figure 4:**
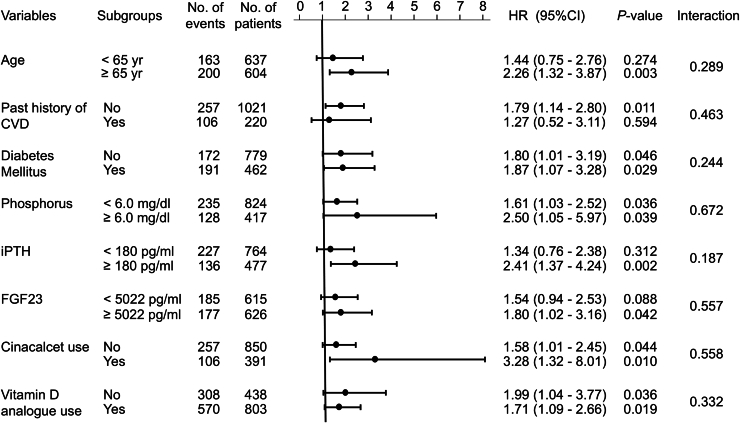
Subgroup analysis of the association between soluble Klotho and CVD events in the fully adjusted model. CI, confidence interval; CVD, cardiovascular disease; FGF23, fibroblast growth factor; HR, hazard ratio; iPTH, intact parathyroid hormone.

Subgroup analyses were conducted to examine the association between sKlotho concentration and CVD events across different patient characteristics, including age, history of CVD, diabetes mellitus, phosphorus levels, iPTH, FGF23, cinacalcet use, and vitamin D analog use (Figure 4). The significant association between sKlotho concentration and CVD events persisted across most subgroups, except in patients with younger age, a history of CVD, lower PTH levels, and lower FGF23 levels. However, there were no significant interactions in these subgroups.

## Discussion

This study demonstrates that low serum sKlotho levels are associated with CVD events, clinical fracture events, and all-cause mortality in patients undergoing hemodialysis. To the best of our knowledge, this is the largest study to date that sufficiently establishes an association between serum sKlotho levels and these outcomes in a hemodialysis population. Moreover, it is the first study to report an association between sKlotho levels and fracture events using a cohort study design.

Several previous studies have explored the relationship between sKlotho levels and clinical outcomes, including mortality and CVD events, in both dialysis^20^^,^^23^, ^24^, ^25^, ^26^, ^27^ and nondialysis patients.^21^^,^^22^^,^^28^, ^29^, ^30^, ^31^ Nowak *et al.*^25^ found that higher sKlotho levels were associated with a reduced risk of atrial fibrillation (HR = 0.66; 95% CI: 0.41–1.00) in patients undergoing dialysis. Similarly, Marcais *et al.*^20^ reported in a prospective cohort study of 238 patients on hemodialysis that serum sKlotho levels > 280 ng/ml were associated with a lower risk of 2-year CVD events (odds ratio = 0.86; 95% CI: 0.76–0.99). Consistent with our findings, studies on patients on dialysis have generally shown that low sKlotho concentrations are associated with increased incidence of CVD and mortality.^20^^,^^24^, ^25^, ^26^, ^27^

Conversely, clinical studies that did not include patients on dialysis have shown controversial results regarding the association between sKlotho and clinical outcomes. Some studies have reported that low sKlotho levels are associated with CVD events in patients with CKD, excluding those on dialysis.^28^^,^^30^ In contrast, many large-scale studies on patients with CKD, not on dialysis, have negative findings regarding sKlotho and outcomes.^21^^,^^22^^,^^29^ In representative research, Edmonston *et al.*^22^ reported that sKlotho was not associated with clinical outcomes from Chronic Renal Insufficiency Cohort study (*n* = 1088; estimated glomerular filtration rate: 42 ml/min per 1.73 m^2^ (HR = 0.77; 95% CI: 0.32–1.89). Possibly, the organ-protective role of sKlotho differs between patients receiving and those not receiving dialysis. sKlotho concentrations are higher in patients not undergoing dialysis; however, reports on patients on dialysis often show low concentrations. In this study, the overall Klotho concentration in patients not undergoing dialysis was lower than that reported in previous studies. Therefore, the protective effect of sKlotho may be more pronounced in patients undergoing dialysis.

Mechanistically, sKlotho may influence CVD events through several mechanisms. First, sKlotho has been shown to protect against uremic cardiomyopathy and cardiac remodeling. In a heterozygous Klotho gene-knockout CKD mouse model, detectable sKlotho concentrations were linked to rapid cardiac hypertrophy and fibrosis.^32^ In addition, the administration of Klotho-encoding transgenes improved cardiomyopathy in Klotho-deficient mice.^9^ Clinically, sKlotho levels have been associated with left ventricular hypertrophy in both patients with CKD^33^ and those on hemodialysis,^34^ suggesting its critical role in preventing cardiac hypertrophy. Second, sKlotho modulates nitric oxide bioavailability in endothelial cells, which is essential for maintaining vascular function. Endothelial dysfunction, a precursor to atherosclerosis, is characterized by reduced nitric oxide bioavailability, impaired vasorelaxation, increased oxidative stress, and increased endothelial permeability.^35^ sKlotho has been shown to enhance vascular protection by regulating nitric oxide availability and reducing aortic and arteriolar vasodilation.^36^ Moreover, sKlotho mitigates the effect of phosphate and FGF23 on aortic contraction through increased nitric oxide production. Third, sKlotho protects against vascular calcification. A previous study reported that sKlotho suppressed phosphate-induced vascular calcification in rat smooth muscle cells.^37^ Finally, sKlotho treatment reduced hyperphosphatemia and prevented vascular calcification in mice lacking endogenous Klotho.^38^ We also found an association between CVD events and sKlotho levels. CVD events greatly contributed to all-cause mortality, but no relationship was observed with the occurrence of infections or cancer in this study (data not shown). Therefore, the main effect of Klotho on all-cause mortality was thought to be CVD.

Our multivariate analysis revealed that the association between sKlotho levels and CVD events persisted even after adjusting for bone mineral markers, such as FGF23; and medications, including phosphate binders, vitamin D analogs, and calcimimetics. No significant interactions were detected in subgroup analyses involving FGF23, phosphorus, PTH, or active vitamin D analogs, suggesting that the effects of sKlotho on CVD were independent of these factors. Previous studies in CKD mouse models have similarly shown that sKlotho cardioprotective effects are independent of phosphate and FGF23.^32^ One proposed mechanism is the downregulation of stress-induced transient receptor potential canonical 6 channels in the heart. Furthermore, recent research indicates that ganglioside-enriched lipid rafts acted as sKlotho receptors, mediating its cardioprotective effects independent of FGF receptor signalling.^39^ Our results reinforce these mechanistic insights and underscore the need for future well-designed studies on sKlotho in CKD patients.

This study is the first to establish an association between sKlotho levels and clinical fracture events in a cohort of patients on dialysis. In animal models, Klotho protein deficiency has been shown to result in low bone turnover, reduced cortical thickness, decreased bone mass, and altered osteocyte distribution.^40^ Studies have demonstrated a link between sKlotho and bone homeostasis, with sKlotho promoting the expression of early growth response protein 1, a regulator of calcium mineralization, chondrogenesis, and osteogenesis.^41^ A small clinical study in diabetes patients found that fractures were more frequent in those with lower skeletal muscle mass.^42^ Given that the number of fractures in our study was small and included clinically insignificant cases (e.g., minor lumbar compression and pelvic fractures), further research is needed to explore the relationship between sKlotho and fracture risk.

A key question is how serum sKlotho levels can be increased. Our study found a significant association between higher sKlotho levels, lower FGF23 levels, and the use of active vitamin D analogs. Supporting this, a small clinical study reported that paricalcitol administration in renal transplant patients increased sKlotho levels and reduced Klotho promoter methylation.^43^ Another study demonstrated that in human renal cells, activation of the vitamin D receptor enhances *Klotho* gene expression.^44^ Thus, strategies such as lowering FGF23 levels and administering active vitamin D preparations may help elevate sKlotho levels. However, because our cross-sectional analysis could not establish the direction of this association, clinical trials are needed to determine whether increasing sKlotho has a therapeutic effect. In this study, the use of cinacalcet was associated with a decrease in sKlotho. It is possible that the effects of cinacalcet users, who are thought to have high levels of phosphorus and PTH, were taken into consideration. Because this multivariate analysis of sKlotho was a cross-sectional analysis, it is difficult to state causality. Previous studies have reported that administration of the ARB, valsartan/hydrochlorothiazide increases sKlotho.^45^ Another report suggested that ARBs have increased sKlotho^46^; however, in this study, the use of angiotensin-converting enzyme inhibitor or ARBs did not correlate with sKlotho. Previous studies targeted patients with conservative renal failure rather than patients with dialysis, and residual renal function may be necessary to determine the relationship between ARBs and sKlotho concentrations. Previous studies have reported a relationship between sKlotho and renal anemia.^47^^,^^48^ In this study, no significant relationship was found between sKlotho and anemia or the use of erythropoietin stimulating agents; however, it is possible that sKlotho is involved in renal anemia and erythropoietin stimulating agent–resistant anemia, and further research is needed to address this option. Recombinant sKlotho protein variants have been developed, and their usefulness has been demonstrated in several basic research studies.^49^^,^^50^ Future developments may enable sKlotho replenishment, making it a potential therapeutic target for CKD patients.^51^

This study has some important limitations. First, as an observational study, it cannot establish a causal relationship between serum sKlotho levels and clinical events. Second, sKlotho levels were not measured immediately after serum collection, and instead, frozen samples were used. Previous studies have reported that using frozen serum does not significantly affect measurement results.^52^^,^^53^ Third, there are various methods for measuring sKlotho; however, it was not possible to compare among them in this study. Whereas this study used IBL's enzyme-linked immunosorbent assay, a recent Chronic Renal Insufficiency Cohort study pointed out the possibility of specifically measuring sKlotho using Quidel’s assay.^22^ Therefore, in the future, it is desirable to conduct research to evaluate sKlotho using multiple assays and investigate its relationship with various clinical events. Fourth, we did not evaluate cardiac function using echocardiography or magnetic resonance imaging, despite evidence that sKlotho may influence cardiac hypertrophy.^9^ Finally, it has been reported that the hormonal activity and total amount of degradation products of intact and c-terminal FGF23 are different, and differences in their impact on various clinical prognoses are also observed.^54^ There are 2 forms of FGF23 that can be detected: intact FGF23 and C-terminal FGF23. Intact FGF23 is considered to possess biological activity, whereas C-terminal FGF23 is believed to be inactive and indicate the total amount of degradation. Regarding the effect on sKlotho, it has been reported that intact FGF23 forms a complex similar to membrane-type Klotho, but the role of the C-terminal has not yet been clarified. Further research is needed to determine how the effects of sKlotho differ between intact and C-terminal FGF23 forms.

In conclusion, this study demonstrates an association between sKlotho levels and CVD events, fracture events, and all-cause mortality in hemodialysis patients. Further well-designed observational and interventional studies are needed to confirm these findings and explore potential therapeutic implications.

## Disclosure

All the authors declared no competing interests.

## Supplementary Material

Supplementary File (PDF) **Figure S1.** Kaplan–Meier cumulative incidence curve of cardiovascular disease events by soluble Klotho concentration. **Figure S2.** Kaplan–Meier cumulative incidence curve of fracture events by soluble Klotho concentration. **Figure S3.** Kaplan–Meier cumulative incidence curve of all-cause mortality by soluble Klotho concentration. **Table S1.** Multivariate regression models predicting serum Klotho concentration (pg/ml). **Table S2.** Cox hazard regression analysis for cardiovascular disease events by serum soluble Klotho concentration. **Table S3.** Cox hazard regression analysis for fracture events by serum soluble Klotho concentration. **Table S4.** Cox hazard regression analysis for all-cause mortality by serum soluble Klotho concentration. STROBE Checklist.
