# Supplementary material for: Klotho’s Impact on Cardiovascular Disease, Fractures, and Mortality in Hemodialysis
Source: Kidney Int Rep. 2025 Jul 30;10(10):3516–26. doi: 10.1016/j.ekir.2025.07.032 (PMC12545757; doi:10.1016/j.ekir.2025.07.032)
Supplement: Supplementary File (PDF) — Figure S1. Kaplan–Meier cumulative incidence curve of cardiovascular disease events by soluble Klotho concentration. Figure S2. Kaplan–Meier cumulative incidence curve of fracture events by soluble Klotho concentration. Figure S3. Kaplan–Meier cumulative incidence curve of all-cause mortality by soluble Klotho concentration. Table S1. Multivariate regression models predicting serum Klotho concentration (pg/ml). Table S2. Cox hazard regression analysis for cardiovascular disease events by serum soluble Klotho concentration. Table S3. Cox hazard regression analysis for fracture events by serum soluble Klotho concentration. Table S4. Cox hazard regression analysis for all-cause mortality by serum soluble Klotho concentration. STROBE Checklist. [file mmc1.pdf]

Supplementary Table S1. Multivariate regression models predicting serum Klotho concentration (pg/mL)

Parameter	Parameter Estimate	Standard error	P value
Age	-1.33	0.60	0.026
Sex (male)	-22.01	13.75	0.11
Body mass index	0.95	1.20	0.426
Diabetes mellitus	-23.60	13.36	0.078
Hemoglobin	7.58	6.06	0.211
ESA	4.30	15.10	0.543
ACE-I or ARB	-23.12	13.19	0.08
Calcium channel blocker	23.23	13.29	0.081
Albumin	-36.62	19.51	0.061
Creatinine	-0.03	2.43	0.99
Phosphate	1.65	4.92	0.737
Calcium	-1.74	10.29	0.866
iPTH	0.04	0.04	0.355
FGF23*1000	-0.90	0.45	0.022
C-reactive protein	-5.15	3.91	0.188
Magnesium	-18.68	13.23	0.158
Active vitamin D analogue	20.24	11.20	0.041
Lanthanum	5.32	13.44	0.692
Sevelamer	7.50	15.51	0.629
Bixsalomer	-3.49	35.47	0.922
CaCO3	9.86	13.64	0.143
Past history of PEIT or PTX	51.62	20.30	0.01
Cinacalcet	-22.61	13.70	0.044

Abbreviations: ESA, erythropoietin stimulating agent; ACE-I, angiotensin converting enzyme inhibitor; ARB, angiotensin receptor blocker; iPTH, intact parathyroid hormone; FGF23, fibroblast growth factor 23; PEIT, percutaneous ethanol injection therapy; PTx, parathyroidectomy

Supplementary Table S2. Cox hazard regression analysis for cardiovascular disease events by serum soluble Klotho concentration

	Quartiles of serum Klotho concentration (pg/mL)				
	Quartile 1 (< 249)	Quartile 2 (249-326)	Quartile 3 (326-434)	Quartile 4 (≥ 434)	*P*-value
Participants (n)	307	310	309	315	
Events (n)	136	116	97	87	<0.001
Unadjusted	1.84 (1.37-2.48)	1.43 (1.06-1.95)	1.15 (0.84-1.59)	Ref	<0.001
Model I	1.78 (1.23-2.57)	1.41 (0.96-2.05)	1.01 (0.68-1.49)	Ref	<0.001
Model II	1.79 (1.22-2.63)	1.44 (0.97-1.47)	0.97 (0.83-1.47)	Ref	<0.001
Model III	1.76 (1.20-2.60)	1.14 (0.95-2.10)	0.96 (0.64-1.45)	Ref	<0.001

Model I: Adjusted for age, sex, dialysis vintage, body mass index, systolic blood pressure, diabetes mellitus, hemoglobin, albumin, creatinine, potassium, C-reactive protein, beta 2 microglobulin, use of angiotensin-converting enzyme (ACE)-inhibitors or angiotensin II receptor blockers, history of cardiovascular disease, history of atrial fibrillation, and Kt/V for multiple regression analysis.

Model II: Model I + (calcium, phosphate, magnesium, intact parathyroid hormone, fibroblast growth factor 23, 25-hydroxyvaitaminD and history of percutaneous ethanol injection therapy or parathyroidectomy)

Model III: Model II + (active vitamin D analog, lanthanum, sevelamer, bixasalomer, $CaCO_3$, and cinacalcet)

Supplementary Table S3. Cox hazard regression analysis for fracture events by serum soluble Klotho concentration

	Quartile of serum Klotho concentration (pg/mL)				
	Quartile 1 (< 249)	Quartile 2 (249-326)	Quartile 3 (326-434)	Quartile 4 (≥ 434)	*P*-value
Participants (n)	307	310	309	315	
Events (n)	30	27	25	18	<0.001
Unadjusted	1.83 (1.01-3.31)	1.65 (0.91-3.01)	1.49 (0.81-2.72)	Ref	<0.001
Model I	1.79 (0.97-3.27)	1.58 (0.77-2.92)	1.76 (0.77-2.70)	Ref	0.58
Model II	1.98 (1.01-3.81)	1.96 (0.99-3.88)	1.79 (0.90-3.57)	Ref	<0.001
Model III	1.99 (1.01-3.91)	2.04 (1.02-4.07)	1.80 (0.91-3.56)	Ref	<0.001

Model I: Adjusted for age, dialysis vintage, history of fracture, and history of percutaneous ethanol injection therapy or parathyroidectomy in multiple regression analysis

Model II: Model I + (albumin, beta 2 microglobulin magnesium, and fibroblast growth factor 23).

Model III: Model II + (intact parathyroid hormone and cinacalcet)

Supplementary Table S4. Cox hazard regression analysis for all-cause mortality by serum soluble Klotho concentration

	Quartile of serum Klotho concentration (pg/mL)				
	Quartile 1 (< 249)	Quartile 2 (249-326)	Quartile 3 (326-434)	Quartile 4 (≥ 434)	*P*-value
Participants (n)	307	310	309	315	
Events (n)	61	70	50	47	<0.001
Unadjusted	1.40 (0.96-2.04)	1.57 (1.08-2.27)	1.09 (0.73-1.62)	Ref	<0.001
Model I	1.40 (0.84-2.34)	1.57 (0.95-2.60)	0.98 (0.58-1.68)	Ref	0.58
Model II	1.59 (0.92-2.73)	1.77 (1.03-3.03)	1.11 (0.63-1.95)	Ref	<0.001
Model III	1.74 (1.00-3.03)	1.92 (1.11-3.36)	1.13 (0.63-2.03)	Ref	<0.001

Model I: Adjusted for age, sex, dialysis vintage, body mass index, systolic blood pressure, diabetes mellitus, hemoglobin, albumin, creatinine, potassium, C-reactive protein, beta 2 macroglobulin, use of angiotensin-converting enzyme (ACE)-inhibitors or angiotensin II receptor blockers, history of cardiovascular disease, history of atrial fibrillation, and Kt/V for multiple regression analysis.

Model II: Model I + (calcium, phosphate, magnesium, intact parathyroid hormone, fibroblast growth factor 23, 25-hydroxyvaitaminD and history of percutaneous ethanol injection therapy or parathyroidectomy)

Model III: Model II + (active vitamin D analog, lanthanum, sevelamer, bixasalomer, $CaCO_3$,and cinacalcet)

Supplementary Figure S1 **Kaplan–Meier cumulative incidence curve of cardiovascular disease events by soluble Klotho concentration**

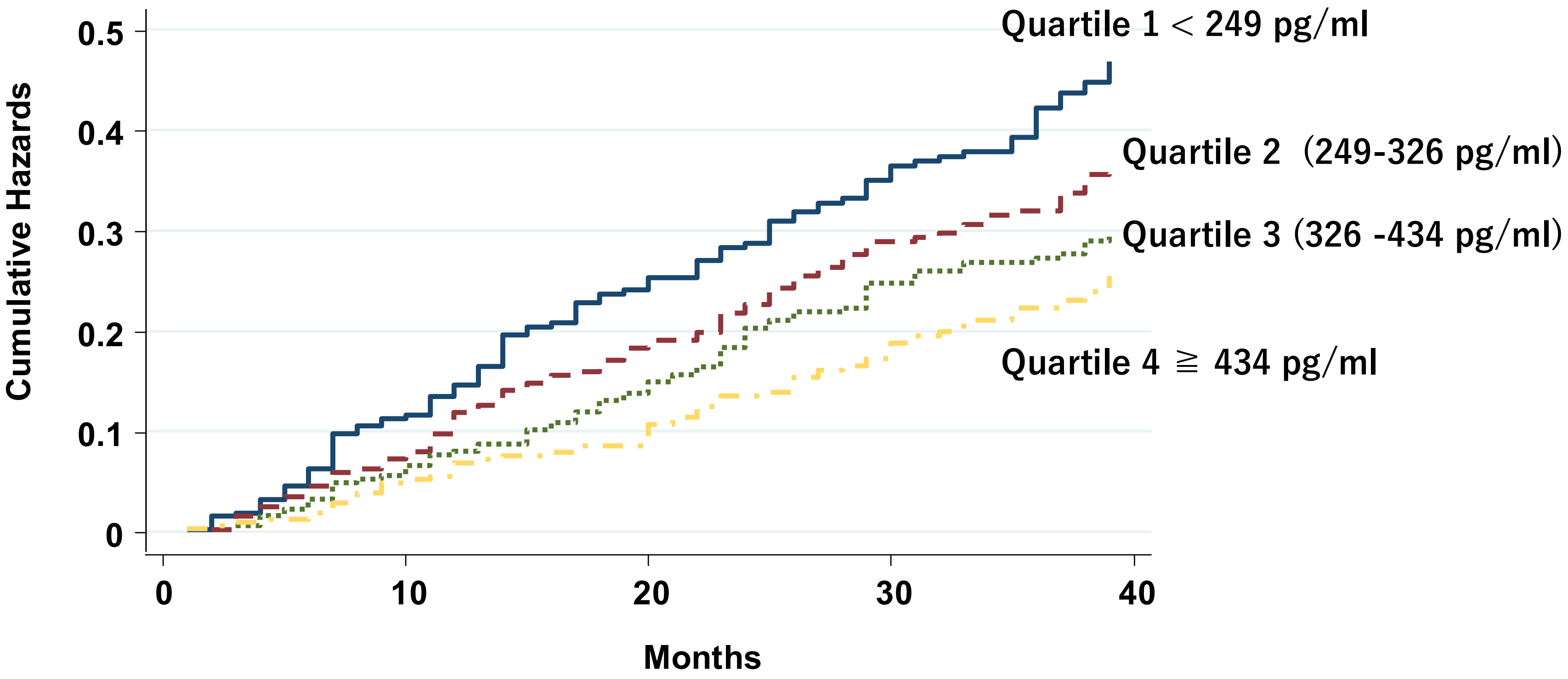

Supplementary Figure S2 Kaplan–Meier cumulative incidence curve of fracture events by soluble Klotho concentration

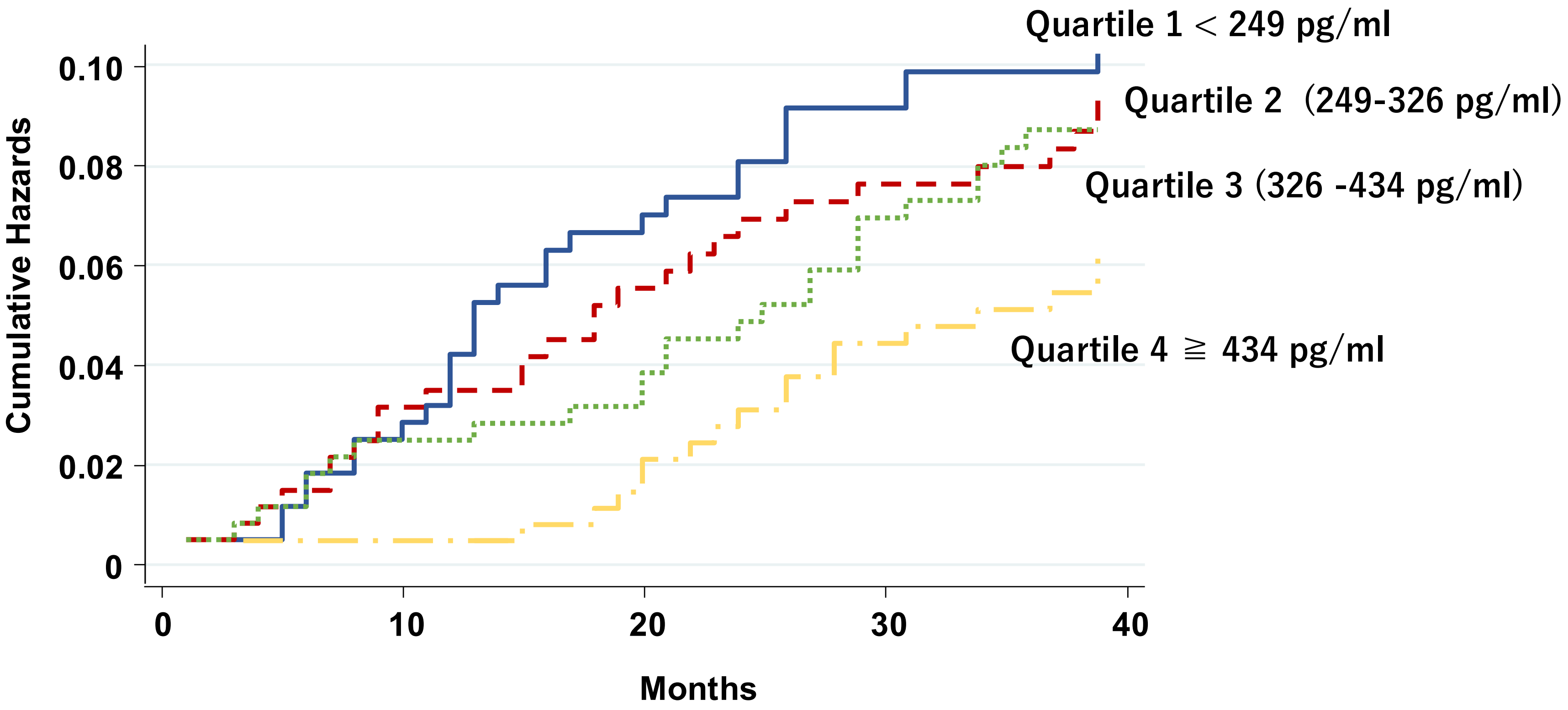

Supplementary Figure S3 **Kaplan–Meier cumulative incidence curve of all-cause mortality by soluble Klotho concentration**

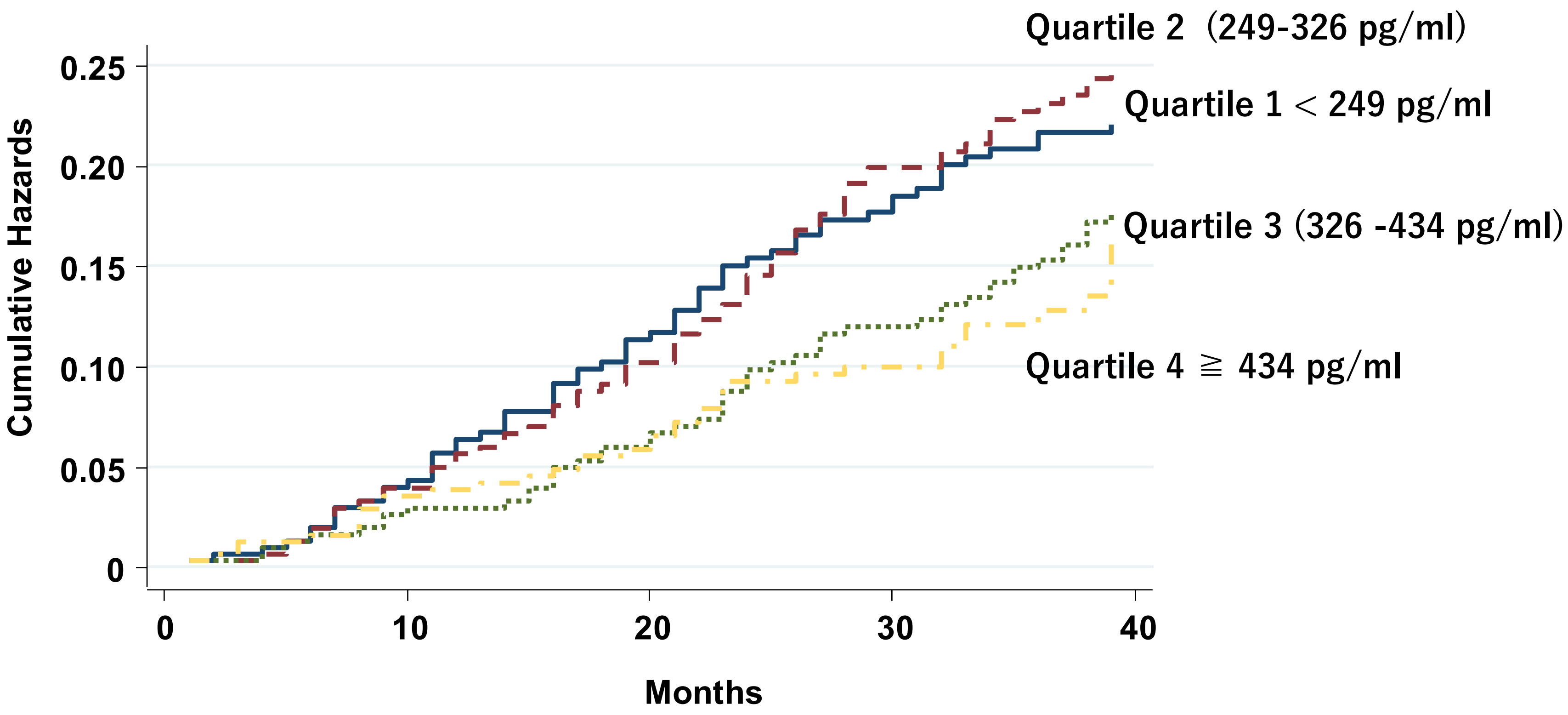

STROBE Statement—checklist of items that should be included in reports of observational studies

	Item No.	Recommendation	Page No.
**Title and abstract**	1	(*a*) Indicate the study's design with a commonly used term in the title or the abstract	P1
		(*b*) Provide in the abstract an informative and balanced summary of what was done and what was found	P2
**Introduction**			
Background/rationale	2	Explain the scientific background and rationale for the investigation being reported	P4-5
Objectives	3	State specific objectives, including any prespecified hypotheses	P5
**Methods**			
Study design	4	Present key elements of study design early in the paper	P5
Setting	5	Describe the setting, locations, and relevant dates, including periods of recruitment, exposure, follow-up, and data collection	P5-6
Participants	6	(*a*) *Cohort study*—Give the eligibility criteria, and the sources and methods of selection of participants. Describe methods of follow-up<br>*Case-control study*—Give the eligibility criteria, and the sources and methods of case ascertainment and control selection. Give the rationale for the choice of cases and controls<br>*Cross-sectional study*—Give the eligibility criteria, and the sources and methods of selection of participants	P5
		(*b*) *Cohort study*—For matched studies, give matching criteria and number of exposed and unexposed<br>*Case-control study*—For matched studies, give matching criteria and the number of controls per case	P5
Variables	7	Clearly define all outcomes, exposures, predictors, potential confounders, and effect modifiers. Give diagnostic criteria, if applicable	P7
Data sources/ measurement	8*	For each variable of interest, give sources of data and details of methods of assessment (measurement). Describe comparability of assessment methods if there is more than one group	P7
Bias	9	Describe any efforts to address potential sources of bias	P8
Study size	10	Explain how the study size was arrived at	N/A

Continued on next page

Quantitative variables	11	Explain how quantitative variables were handled in the analyses. If applicable, describe which groupings were chosen and why	P8
Statistical methods	12	(*a*) Describe all statistical methods, including those used to control for confounding	P8-9
		(*b*) Describe any methods used to examine subgroups and interactions	P8-9
		(*c*) Explain how missing data were addressed	P8-9
		(*d*) *Cohort study*—If applicable, explain how loss to follow-up was addressed<br>*Case-control study*—If applicable, explain how matching of cases and controls was addressed<br>*Cross-sectional study*—If applicable, describe analytical methods taking account of sampling strategy	P8-9
		(*e*) Describe any sensitivity analyses	P8-9
Participants	13*	(a) Report numbers of individuals at each stage of study—eg numbers potentially eligible, examined for eligibility, confirmed eligible, included in the study, completing follow-up, and analysed	P9-10
		(b) Give reasons for non-participation at each stage	P9
		(c) Consider use of a flow diagram	N/A
Descriptive data	14*	(a) Give characteristics of study participants (eg demographic, clinical, social) and information on exposures and potential confounders	P9
		(b) Indicate number of participants with missing data for each variable of interest	P9
		(c) *Cohort study*—Summarise follow-up time (eg, average and total amount)	P9-10
Outcome data	15*	*Cohort study*—Report numbers of outcome events or summary measures over time	*P10-13*
		Case-control study—Report numbers in each exposure category, or summary measures of exposure	N/A
		Cross-sectional study—Report numbers of outcome events or summary measures	N/A
Main results	16	(*a*) Give unadjusted estimates and, if applicable, confounder-adjusted estimates and their precision (eg, 95% confidence interval). Make clear which confounders were adjusted for and why they were included	P10-12
		(*b*) Report category boundaries when continuous variables were categorized	P10-12
		(*c*) If relevant, consider translating estimates of relative risk into absolute risk for a meaningful time period	

Continued on next page

Other analyses	17	Report other analyses done—eg analyses of subgroups and interactions, and sensitivity analyses	P10-12
Key results	18	Summarise key results with reference to study objectives	P13
Limitations	19	Discuss limitations of the study, taking into account sources of potential bias or imprecision. Discuss both direction and magnitude of any potential bias	P17-18
Interpretation	20	Give a cautious overall interpretation of results considering objectives, limitations, multiplicity of analyses, results from similar studies, and other relevant evidence	P18
Generalisability	21	Discuss the generalisability (external validity) of the study results	P18
**Other information**			
Funding	22	Give the source of funding and the role of the funders for the present study and, if applicable, for the original study on which the present article is based	P19

*Give information separately for cases and controls in case-control studies and, if applicable, for exposed and unexposed groups in cohort and cross-sectional studies.

Note: An Explanation and Elaboration article discusses each checklist item and gives methodological background and published examples of transparent reporting. The STROBE checklist is best used in conjunction with this article (freely available on the Web sites of PLoS Medicine at http://www.plosmedicine.org/, Annals of Internal Medicine at http://www.annals.org/, and Epidemiology at http://www.epidem.com/). Information on the STROBE Initiative is available at www.strobe-statement.org.
